# Clinical and Economic Implications of High-Sensitivity Troponin-Informed Admission Strategies in Non-AMI Chest Pain

**DOI:** 10.3390/jcdd13070328

**Published:** 2026-07-14

**Authors:** Wanyi Chen, Allan S. Jaffe, Fred S. Apple, Christopher deFilippi, William Frank Peacock, Alan H. B. Wu, Rana Fayyad, Sarah Bethoney, Jingjing Zhang, Artem T. Boltyenkov

**Affiliations:** 1 Medical Affairs, Siemens Healthcare Diagnostic Inc., Tarrytown, NY 10591, USAartem.boltyenkov@siemens-healthineers.com (A.T.B.); 2 Cardiovascular Department and Department of Laboratory Medicine and Pathology, Mayo Clinic, Rochester, MN 55905, USA; 3 Department of Laboratory Medicine and Pathology, Hennepin Healthcare/Hennepin County Medical Center, Minneapolis, MN 55404, USA; 4 Hennepin Healthcare Research Institute, Minneapolis, MN 55404, USA; 5 School of Medicine, University of Maryland, Baltimore, MD 21201, USA; 6 Emergency Department, Ben Taub Hospital, Baylor College of Medicine, Houston, TX 77030, USA; 7 Department of Laboratory Medicine, University of California, San Francisco, CA 94143, USA

**Keywords:** high-sensitivity troponin, prognostic value, economic evaluation, chest pain, acute myocardial infarction

## Abstract

Most patients with chest pain do not have acute myocardial infarction (AMI), yet post-AMI rule-out disposition remains variable. Detectable but sub-99th percentile high-sensitivity cardiac troponin I (hs-cTnI) provides additional prognostic information. We evaluated the economic value of hs-cTnI-guided hospitalizations among non-AMI patients. We analyzed 1481 non-AMI chest pain visits with detectable baseline hs-cTnI across 29 U.S. emergency departments (2014–2016) in the prospective HIGH-US trial using the Atellica IM TnIH assay(Siemens Healthcare Diagnostics, Tarrytown, NY, US). We compared observed standard-of-care admissions with modeled pathways incorporating hs-cTnI thresholds and risk scores, assessing admission rates, costs, and diagnostic accuracy for 30-day death/MI. Overall, 1.0% (*n* = 15/1481) experienced 30-day death/MI. Standard-of-care admitted 59% of patients with 93% sensitivity and 41% specificity. The modeled pathway using hs-cTnI ≥ 5 ng/L plus non-low risk scores was associated with reduced projected admission of 41%, higher specificity of 60%, and the same 93% sensitivity. Estimated per-patient diagnostic costs were lower under Scenario 1 (noninvasive diagnostic testing only; $1025 vs. $1139) and under Scenario 2 (including inpatient/invasive procedures; $2672 vs. $3536). In subgroups, higher hs-cTnI thresholds conferred further economic benefit without compromising sensitivity. Incorporating sub-99th percentile hs-cTnI thresholds alongside risk scores may support more efficient resource use while maintaining safety, although findings require prospective validation.

## 1. Introduction

Chest pain is among the most common reasons for emergency department (ED) visits in the United States, accounting for over 10 million annual encounters for suspected acute coronary syndromes (ACSs) [1,2] and generating $5–10 billion dollars in direct medical costs [3,4]. Early diagnosis and effective risk stratification in this population, who are at risk of major adverse cardiac events (MACEs, defined as death, acute myocardial infarction [AMI], heart failure [HF] hospitalization, or an urgent revascularization procedure of percutaneous coronary intervention or coronary artery bypass grafting), remain central priorities in emergency medicine.

High-sensitivity cardiac troponin (hs-cTn) is a cornerstone biomarker in cardiovascular care, widely recognized for its role in the rapid diagnosis of AMI [5,6]. Current U.S. and European guidelines endorse use of the assay-specific 99th percentile upper reference limit (URL) as the primary rule-in cutoff for AMI, interpreted in conjunction with serial changes and clinical assessment [6,7]. Using these criteria, more than 90% of chest pain presentations ultimately have AMI ruled out [8]. Among these patients, short- and long-term MACE risk varies, and disposition decisions after AMI exclusion remain variable across clinical settings. Balancing the risk of missed events against potentially avoidable hospitalizations continues to be an important operational challenge.

Evidence suggests that hs-cTnI concentrations below the 99th percentile URL, particularly values above the limit of detection (LoD), are associated with higher rates of adverse outcomes compared with undetectable levels [9,10,11,12,13]. When combined with clinical risk scores integrating ECG findings and cardiac risk factors, hs-cTnI further enhances risk discrimination and refines the identification of patients suitable for early discharge [2,3,14].

Despite these advances, less is known about how sub-99th percentile hs-cTn values, particularly among patients with detectable concentrations, can be incorporated into admission decisions after AMI has been ruled out. Most contemporary pathways focus on identifying very-low-risk patients for discharge, while variability persists in managing patients with intermediate levels of risk [15,16,17]. There is limited evidence quantifying how alternative admission strategies informed by hs-cTn may affect resource use relative to current practice.

To address this evidence gap, we conducted a secondary analysis of the HIGH-US study, which prospectively enrolled 2505 suspected ACS patients presenting to 29 U.S. EDs [18,19]. Our objective was to evaluate the prognostic and economic value of incorporating sub-99th percentile hs-cTnI with established risk scores to guide admission decisions among patients with detectable hs-cTnI and in whom AMI has been ruled out.

## 2. Materials and Methods

### 2.1. Overview of HIGH-US Study

We conducted a secondary analysis of the HIGH-US trial, which prospectively enrolled 2499 adults (≥22 years) presenting with suspected ACS to 29 U.S. EDs between 04/2015 and 04/2016 [18,19]. All participants provided written informed consent, and institutional review boards at participating centers approved the study. Demographics, cardiac risk factors, medical history, concurrent therapies, physician’s pre-troponin suspicion of ACS, and all ECG findings were recorded.

#### 2.1.1. Troponin I Measurement

Baseline blood samples were obtained at a median of 93 min (IQR: 68–122) after ED arrival, with additional samples collected at 1 and 3 h. Hs-cTnI was measured using the Atellica IM TnIH assay (Siemens Healthcare Diagnostics, Tarrytown, NY, USA); limit of blank: 0.50 ng/L; limit of detection: 1.60 ng/L; limit of quantitation: 2.50 ng/L; coefficient of variation: ≤10%; pooled 99th percentile: 45 ng/L) [18,20].

#### 2.1.2. HEAR Score

HEAR (History, ECG, Age, Risk factors) score elements are detailed in Supplementary Table S1. Individual HEAR components were obtained through structured medical record review and interviews with patients and treating physicians blinded to hs-cTnI results. The score was modified as described previously [21]. Consistent with the prior literature, a predefined cutoff ≥ 4 defined non-low-risk patients [22,23,24,25].

#### 2.1.3. Outcomes

Index AMI was adjudicated by two independent reviewers using local contemporary cardiac troponin assays according to the Third Universal Definition of Myocardial Infarction, with a third adjudicator resolving discrepancies [26]. Participants were prospectively followed for all-cause mortality and MACE through 365 days. MACE was defined as AMI, cardiac death, heart failure hospitalization, or urgent revascularization procedure (percutaneous coronary intervention or coronary artery bypass grafting). The present analysis focuses on 30-day death or MI.

#### 2.1.4. Final Analytic Cohort

**Figure 1:**
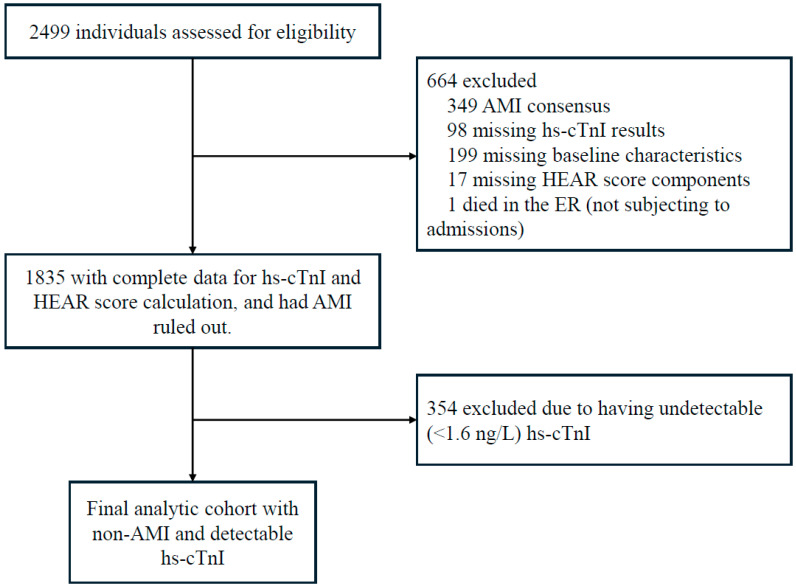
Consort diagram for enrolled patients and patients included in the final analysis.

To focus on the prognostic (rather than diagnostic) utility of hs-cTnI, we excluded patients with an adjudicated index AMI and those with missing hs-cTnI measurements or HEAR score components, yielding 1835 patients (Figure 1). Within this non-AMI cohort, the median baseline hs-cTnI concentration was 5 ng/L (IQR: 2–14); 19% (n = 354) had undetectable hs-cTnI values (<1.6 ng/L), and 9.5% (n = 175) had concentrations exceeding the 99th percentile. Because our objective was to evaluate the prognostic and economic implications of integrating detectable sub-99th percentile hs-cTnI into admission decisions among patients, we further excluded individuals with undetectable troponin values, yielding a final analytic cohort of 1481 patients (Figure 1). Baseline characteristics are shown in Table 1. Within this final analytic cohort, 15 experienced death or MI within 30 days.

### 2.2. Hospital Admission Pathways

We modeled hypothetical hospital admission pathways integrating HEAR scores with presenting hs-cTnI concentrations and compared them against observed standard-of-care (SOC) admissions in the HIGH-US trial.

Candidate hs-cTnI thresholds were prespecified using distributional landmarks from the 1835-patient non-AMI cohort prior to exclusion of patients with undetectable hs-cTnI. The median and third-quartile hs-cTnI concentrations in this cohort were 5 ng/L and 14 ng/L, respectively. Thresholds of 4–6 ng/L and 13–15 ng/L were evaluated around these landmarks. Following a tertiary approach, additional thresholds of 20 ng/L, 29 ng/L, and the pooled 99th percentile (45 ng/L) were also evaluated.

This yielded nine modeled pathways in which admission was recommended when both HEAR ≥ 4 and baseline hs-cTnI exceeded a prespecified threshold (4, 5, 6, 13, 14, 15, 20, 29, or 45 ng/L). Patients not meeting these criteria were assumed to receive ED-based management. For each pathway, we evaluated admission rates, diagnostic performance for 30-day death or MI, and per-patient costs. To assess the incremental contribution of hs-cTnI beyond clinical risk assessment alone, we additionally evaluated a HEAR-only strategy (admit if HEAR ≥ 4).

### 2.3. Cost Analysis

We estimated per-patient direct medical costs from the U.S. healthcare payer perspective over the index encounter. Because patient-level Diagnosis-Related Group (DRG) assignments and detailed timing of diagnostic testing were unavailable, two costing scenarios were evaluated.

*Scenario 1: noninvasive diagnostic testing only.* Costs were limited to noninvasive diagnostic testing, assuming all tests occurred in the outpatient setting. Services were valued using national Medicare Physician Fee Schedule and Outpatient Prospective Payment System reimbursement rates mapped to corresponding Current Procedural Terminology (CPT) code(s). This scenario isolates differences in diagnostic testing intensity independent of inpatient facility payments.

*Scenario 2: inclusion of hospital admission and invasive procedures.* In addition to Scenario 1 costs, inpatient admissions and invasive procedures were included. Hospitalization costs were estimated using national average Medicare Severity Diagnosis-Related Group payments. In the absence of patient-level DRG codes, categories were inferred from observed invasive procedure utilization (PCI, coronary angiography without PCI, or no invasive procedure). Physician professional fees were included separately. To avoid double counting, outpatient facility payments for services assumed to occur during hospitalization were excluded. Costs are reported in 2026 U.S. dollars without discounting due to our chosen short-term focus.

### 2.4. Resource Utilization Estimation

Resource utilization frequencies were derived directly from the study cohort and stratified by observed discharge disposition: (1) ED-based care (including discharge from the ED or an observation unit) and (2) hospital admission. Under modeled pathways, patients assigned to each disposition were assumed to incur the utilization patterns observed among corresponding patients in the HIGH-US cohort. In Scenario 2, hospitalized patients additionally incurred inpatient facility costs. Detailed utilization frequencies, unit costs, and costing algorithms are provided in Table 2 and Table 3.

### 2.5. Subgroup Analysis

To assess the robustness of hs-cTnI-integrated admission pathways across clinically relevant subpopulations, subgroup analyses were performed by hs-cTnI status (detectable/sub-99th percentile vs. above the 99th percentile), sex, age (≥45 years, ≥65 years), and cardiovascular risk factors (diabetes, hypertension, dyslipidemia, obesity, and prior coronary artery disease). Within each subgroup, admission rates, diagnostic performance, and per-patient costs were re-estimated under each modeled pathway relative to SOC.

### 2.6. Statistical Analysis

For each modeled admission pathway, diagnostic performance for 30-day death or MI was assessed using sensitivity, specificity, and negative predictive value (NPV). Sensitivity was defined as the proportion of patients experiencing 30-day death or MI who would have been admitted under the modeled pathway, whereas specificity was defined as the proportion of patients without 30-day death or MI who would not have been admitted. NPV was defined as the proportion of non-admitted patients who remained free of 30-day death or MI.

We present baseline population characteristics as mean ± standard deviation (SD) for continuous variables and percentages (%) for categorical variables (e.g., presence of hypertension). In addition, we compared baseline population characteristics between patients with and without 30-day death/MI using *t* tests for normally distributed continuous variables, Wilcoxon rank sum tests for non-normally distributed continuous variables, and chi-squared tests for categorical variables. We consider *p*-values < 0.05 as statistically significant. *R* software (version 4.5.0) was used for statistical analysis [27].

## 3. Results

Standard-of-care (observed admission decisions). In the HIGH-US trial, the observed admission rate was 59% (879/1481) among patients without an index AMI diagnosis and had detectable hs-cTnI (Table 4). Among the 15 patients who experienced a 30-day death/MI, fourteen were admitted, yielding a sensitivity of 93% for 30-day death/MI under SOC.

Table 5 summarizes the characteristics of the individual (34-year-old male, HEAR = 2, 0 h hs-cTnI = 21.76 ng/L) with a 30-day death/MI (non-cardiac death) who was discharged directly from the ED without additional diagnostic testing or procedures. Under SOC, the NPV for 30-day death/MI was very high (99.8%, 601/602), whereas specificity was modest (41%, 601/1466), reflecting substantial hospitalizations among patients who did not experience adverse events. The estimated per-patient cost was $1139 under Scenario 1 (including cost of noninvasive diagnostic testing only) and increased to $3536 with Scenario 2, which additionally accounted for inpatient facility and procedural fees.

Admission pathways using hs-cTnI and/or HEAR score. Compared with SOC, all hypothetical admission pathways based on elevated baseline hs-cTnI and a non-low HEAR score decreased admission rates to 9–46% (*p* < 0.05) and reduced per-patient costs to $828–$1057 (Scenario 1, *p* < 0.05) and $1177–$2914 (Scenario 2, *p* < 0.05). These pathways also increased specificity for 30-day death/MI to 55–91% (*p* < 0.05) while maintaining a high NPV ≥ 99.1% (Table 4 and Table S2).

When a baseline hs-cTnI threshold at or below the cohort median (≤5 ng/L) was combined with a non-low HEAR score to guide admission, sensitivity for 30-day death/MI was preserved at 93% (14/15), matching SOC performance. With higher cutoff values for hs-cTnI, however, sensitivity would decrease below 93%, indicating that some patients with 30-day death/MI that were admitted under SOC would not have been hospitalized under these more restrictive pathways. In contrast, the counterfactual admission pathway based solely on a non-low HEAR score resulted in admission rates, cost, and diagnostic performance similar to SOC, suggesting that incorporation of hs-cTnI into the admission decision was the main driver of efficiency gains.

Subgroup analyses. Across all clinically relevant subgroups evaluated, the pathway incorporating a 5 ng/L baseline hs-cTnI threshold with HEAR ≥ 4 consistently reduced admission rates and per-patient costs compared with SOC, while preserving sensitivity for 30-day death/MI and maintaining high NPVs (≥99%) (Supplementary Figures S1–S4, Supplementary Table S3). In selected subgroups, including men, patients <65 years, and those with diabetes or obesity, higher hs-cTnI cutoffs up to 13 ng/L further reduced hospitalization and costs without compromising sensitivity.

Among those with baseline hs-cTnI above the 99th percentile, applying an additional HEAR ≥ 4 criterion identified all three individuals with 30-day death/MI, while modestly reducing admissions from 132 under SOC to 128, a difference that was not statistically significant. These findings indicate that the principal prognostic and economic benefit of hs-cTnI-guided admission came from improved discrimination among patients with detectable but normal (below 99th percentile) troponin values.

## 4. Discussion

In this secondary analysis of the prospective HIGH-US trial, we evaluated whether high-sensitivity troponin (hs-cTnI) values below the 99th percentile could be incorporated into admission decision frameworks among patients presenting with non-AMI chest pain and detectable troponin levels. Modeled strategies incorporating a baseline hs-cTnI concentration above the cohort median and a non-low risk score were associated with lower projected hospitalizations compared with observed practice, while preserving sensitivity for 30-day death or MI. In the U.S., over 9 million annual chest pain visits continue to strain ED capacity. Although accelerated diagnostic pathways have transformed AMI rule-in and rule-out, disposition decisions remain variable and conservative for the large patient population in whom AMI is excluded [28,29]. Our findings suggest that incorporating sub-99th percentile hs-cTnI into admission frameworks may support more risk-aligned and economically efficient care in this population.

Importantly, the reduction in hospitalization did not degrade discrimination for 30-day death or MI, as sensitivity and negative predictive value were maintained relative to current practice. While our analysis does not establish that outcomes would be identical under prospective implementation, the modeled strategies identified a sizeable group of patients with low observed 30-day event rates who are nonetheless frequently hospitalized under existing care, despite limited evidence that inpatient admission itself improves short-term outcomes in this population [30,31]. By enhancing specificity in this large population with appreciable risk, an hs-cTn-informed approach may enable more proportionate alignment between patients’ risk and intensity of inpatient resource utilization, rather than presuming hospitalization to be inherently protective.

Subgroup analyses supported the robustness of our findings across clinically relevant strata. In selected populations, including individuals with diabetes or obesity, higher hs-cTnI thresholds (up to 13 ng/L) combined with a non-low risk score were associated with additional projected reductions in hospitalizations without compromising discrimination for 30-day death or MI. This likely reflects the association between subclinically elevated hs-cTn concentrations and an underlying comorbidity burden, suggesting potential value in tailoring troponin thresholds to patient characteristics rather than applying uniform cutoffs [32]. Most of the projected economic benefit arose from improved stratification of patients with detectable but sub-99th percentile hs-cTnI concentrations. This intermediate-risk group appears to offer the greatest opportunity for improving admission efficiency.

Notably, comparison of a counterfactual admission strategy based solely on a non-low risk score with standard-of-care indicates that hs-cTnI was the principal contributor to efficiency gains in hs-cTnI-integrated pathways. This finding aligns with growing literature demonstrating that, in the era of high-sensitivity assays, much of the prognostic discrimination in chest pain evaluation is captured by troponin, with traditional clinical risk scores providing more modest incremental value [33,34]. Our results enforce that hs-cTnI may serve as the dominant quantitative anchor for disposition decisions, with clinical risk scores refining interpretation at the margins.

Emerging evidence suggests that “normal” hs-cTn concentrations (<99th percentile) carry gradations of downstream cardiovascular risks, expanding interest in their role in risk stratification beyond AMI diagnoses [33,35,36]. Because patients with undetectable hs-cTnI consistently exhibit very low short-term event rates, our analysis focused on the clinically relevant population with measurable troponin in whom residual risk, and thus disposition uncertainty, persists [33,37]. Despite growing recognition of this prognostic continuum, the economic implications of operationalizing subclinical hs-cTn elevations to inform resource allocation in emergent chest pain care have received little attention. Our study contributes to this evolving literature by translating sub-99th percentile troponin risk into a pragmatic admission framework and quantifying the potential impact on hospitalization rates and short-term costs. Although the algorithm evaluated here is simplified and retrospectively applied, it serves as a proof-of-concept that structured incorporation of hs-cTnI concentrations below conventional diagnostic cutoffs may inform more risk-aligned and economically efficient admission strategies. Prospective validation will be required to determine how such approaches perform in routine clinical practice.

This study adds to a limited body of literature examining the role of hs-cTn beyond AMI diagnosis by focusing on its potential use in informing admission decisions among patients in whom AMI has been excluded. Prior work has focused on their diagnostic utility for AMI rule-out and their incremental value relative to conventional troponin [38,39,40,41,42,43]. In contrast, the current work shifts the emphasis from diagnosis to prognosis, leveraging hs-cTn to risk-stratify the vast majority who do not meet the criteria for AMI. Another layer of novelty lies in grounding our comparison in observed clinician behavior rather than conventional cTn. Contemporary troponin has been standard practice since 2010 and was the prevailing strategy at the time of the HIGH-US trial [18,44]; accordingly, our comparator reflects the current care environment rather than an outdated paradigm. Moreover, we complement prior secondary analyses of the HIGH-US trial, which primarily examined hs-cTn-guided early discharge [3,17]. Unlike early discharge, non-admission may still involve ED-based observation and additional testing but generally entails less intensive resource utilization than inpatient admission. By quantifying the economic impact of hs-cTn-informed admission decisions, we extend the literature beyond discharge timing to the broader question of aligning patient risk with appropriate resource intensity.

Current chest pain evaluation in the U.S. involves more than 8 million stress tests and cardiac imaging annually, largely driven by efforts to exclude ACS with high diagnostic certainty. This testing-intensive landscape contributes substantially to healthcare expenditures, ED crowding, and downstream resource utilization. Growing evidence suggests that over-investigation confers little benefit in many patients, particularly those with low to moderate risk. Accordingly, guidelines now discourage routine noninvasive cardiac testing in low-risk patients, reflecting real-world observations of little benefit and potential harm. Our findings add to an expanding initiative aimed at modernizing chest pain care, alongside innovations such as emergency-medical-services-based risk stratification and prehospital point-of-care troponin testing. Together, these approaches reflect a broader shift toward earlier, risk-informed decision-making that seeks to match care intensity to patient risk across the continuum of evaluation.

The economic benefit of hs-cTnI-guided admission strategies depends on timely result availability. Although some hospitals achieve turnaround times of ≤60 min, many facilities experience delays due to limited on-site laboratory capacity or reliance on centralized testing. Such delays reduce the utility of troponin for real-time triage and may constrain the adoption of prognostic-based pathways in these settings. Expanding access to rapid laboratory processing or validated point-of-care hs-cTn assays will therefore be critical to ensure that the potential system-level benefits of risk-aligned admission strategies are realized across diverse practice environments, including rural and resource-limited hospitals.

Our study has several limitations. First, the hs-cTnI-based admission criteria were retrospectively applied. One cannot determine whether the modeled disposition decisions would have influenced clinical outcomes under prospective implementation. Second, emergency department workflows, admission practices, laboratory turnaround times, and reimbursement mechanisms vary substantially across institutions and countries. Consequently, the projected reductions in hospitalization and associated cost savings may not be directly generalizable to other healthcare systems. Prospective validation in diverse healthcare environments is therefore important to determine the reproducibility and generalizability of these findings. Third, due to data constraints, we conservatively assumed that all noninvasive diagnostic testing occurred in an outpatient setting, which may underestimate costs. Fourth, in the absence of patient-level DRG codes, we inferred inpatient facility payments based on utilization of invasive procedures; although the impact of this approximation is likely modest, some misclassification is possible. Fifth, we assumed that resource utilization intensity observed in the HIGH-US cohort would mirror those under modeled dispositions. This approach may not be generalizable to other practice environments. Sixth, the HIGH-US trial was conducted between 2015 and 2016 and may not fully reflect contemporary practice patterns. Seventh, we excluded patients with undetectable hs-cTnI, whose short-term event rates are consistently very low and for whom admission decisions may rely on factors beyond troponin concentration. Accordingly, our findings apply specifically to patients with measurable troponin in whom AMI has been ruled out and may not generalize to very-low-risk populations. Lastly, although all hs-cTnI-based pathways maintained NPVs > 99% for 30-day death/MI, the small number of events limits precision. Ultimately, a pragmatic randomized implementation trial is necessary to confirm both safety and economic benefit [45]. In conclusion, among patients presenting with chest pain in whom AMI has been excluded and hs-cTnI is detectable, sub-99th percentile hs-cTnI concentrations provide additional risk information that may help inform admission decisions when considered alongside clinical risk scores. Modeled strategies suggest the potential for more selective use of hospital resources while maintaining sensitivity for short-term adverse events. These findings should be interpreted as hypothesis-generating and require prospective validation in contemporary practice.

## Figures and Tables

**Table 1:** Baseline characteristics of the final analytic cohort of patients without index AMI, with detectable troponin concentrations, and without missing data relevant to this analysis (n = 1481).

	All Patients (n = 1481)	Death or MI Within 30 Days		*p*-Value
		No (n = 1466)	Yes (n = 15)	
Age, years, median (IQR)	57 (50–67)	57 (50–67)	65 (62–68)	*
Male sex, n (%)	880 (59)	868 (59)	12 (80)	>0.05
Race, n (%)				>0.05
White	801 (54)	790 (54)	11 (72)	
Black	626 (42)	623 (42)	3 (20)	
Other	54 (4)	53 (4)	1 (8)	
Risk factors, n (%)				
Obesity	708 (48)	702 (48)	6 (40)	>0.05
Hypertension	1094 (74)	1083 (74)	11 (73)	>0.05
Diabetes	460 (31)	451 (31)	9 (60)	>0.05
Current smoker	378 (26)	376 (26)	2 (13)	>0.05
History, n (%)				
Prior MI	323 (22)	316 (22)	7 (47)	*
Prior revascularization	453 (31)	445 (30)	8 (53)	>0.05
Prior HF hospitalization	355 (24)	348 (24)	7 (47)	>0.05
Coronary artery disease	600 (41)	592 (40)	8 (53)	>0.05
Baseline hs-cTnI, ng/L Median (IQR)	7 (3.5–18.7)	7 (3.5–18.0)	22 (13–32)	*

Abbreviations: HF, heart failure; hs-cTnI, high-sensitivity troponin I; IQR, interquartile range; MI, myocardial infarctions. * Statistically significant at the 0.05 level.

**Table 2:** Detailed breakdown of the cost components, cost values, associated CPT/DRG codes and source, and frequency of resource utilization stratified by patients’ discharge disposition: ED-based care versus hospitalization.

		Frequency of Resource Utilization ^(c)^, %		
Outpatient ^(a)^ Payment	Cost ^(b)^, $, Average (Range)	ED-Based Care	Hospitalization	CPT Code(s)
ED visit	187	100	100	99285
Blood draw	50	319	442	84484
ECG	12 (8–17)	150	184	93000,05,10
Chest X-ray	144 (125–171)	93	96	71045–48
Echocardiogram	875 (859–925)	6	41	93303,06,12
Exercise stress test	80	5	3	93015
Myocardial perfusion imaging	771 (680–862)	9	22	78472–73,81,83
Stress echocardiography	862 (828–895)	8	7	93350–51
Computed tomography coronary angiography	774 (687–823)	9	7	75571–74
Coronary angiography	959	1	19	93454
		**Frequency of resource utilization, %**		
**Inpatient ^(d)^ payment**	**Cost ^(e)^, $,** **Average (range)**	**ED-based care**	**Hospitalization**	**DRG code(s)**
Percutaneous coronary intervention	10,884	-	6	250–251
Chest pain	3586	-	94	313

^(a)^ Outpatient payments included the cost of the ED visit itself and all noninvasive diagnostic tests, which were assumed to have happened in an outpatient setting in this study. ^(b)^ Source: national Medicare Physician Fee Schedule payments and Outpatient Prospective Payment System facility payments mapped to the corresponding CPT code(s). ^(c)^ Estimated based on the observed resource utilization frequency stratified by actual disposition decisions in the study cohort. A value greater than 100% entails that a resource (e.g., ECG) was used multiple times. ^(d)^ Inpatient payments included cost of the admission inferred based on the invasive procedures received. ^(e)^ Source: national Medicare Severity Diagnosis-Related Group payments.

**Table 3:** Algorithms for calculating costs for patients admitted to the hospital and patients managed in the ED or ED-linked observation unit.

	Per-Patient Cost, $
Both Scenario 1 and Scenario 2	
Non-admitted patient	775 = 100% × ED + 319% × Blood + 150% × ECG + 93% × Xray + 6% × Echo + 5% × Exercise + 9% × MPI + 8% × Stress + 9% × CTCA + 1% × CA.
Scenario 1 only	
Admitted patient	1388 = 100% × ED + 442% × Blood + 184% × ECG + 96% × Xray + 41% × Echo + 3% × Exercise + 22% × MPI + 7% × Stress + 7% × CTCA + 19% × CA.
Scenario 2 only	
Admitted patient	5427 = 100% × ED + 442% × Blood + 184% × ECG + 96% × Xray + 41% × Echo + 3% × Exercise + 22% × MPI + 7% × Stress + 7% × CTCA + 19% × CA + 6% × PCI + 94% × non-PCI.

Abbreviations: Blood, cost of a single blood draw for hs-cTnI; CA, cost of a single coronary angiography; CTCA, cost of a single computed tomography coronary angiography; ECG, cost of a single ECG; ED, cost of an ED visit; Echo, cost of a single echocardiography; Exercise, cost of an exercise stress test; MPI, cost of a single myocardial perfusion imaging; non-PCI, inpatient admission cost without a percutaneous coronary intervention; PCI, inpatient admission cost if percutaneous coronary intervention was performed; Stress, cost of a single stress echocardiography; Xray, cost of a single chest X-ray.

**Table 4:** Comparison of admission rates, diagnostic accuracy, and costs by strategy for the final analytic cohort of patients without index AMI, with detectable hs-cTnI, and with no missing data relevant to this analysis (n = 1481)*,* using 30-day death or MI as the adjudicator.

						Cost per Patient, $	
Admission Criteria		% Admitted (n/1481)	NPV, % (n/N)	Sensitivity, % (n/15)	Specificity, % (n/1466)	Scenario 1	Scenario 2
1	0 h hs-cTnI ≥ 4 & HEAR ≥ 4	46% (681)	99.9 (799/800)	93 (14)	55 (799)	1057	2914
2	0 h hs-cTnI ≥ 5 & HEAR ≥ 4	41% (604)	99.8 (876/877)	93 (14)	60 (876)	1025	2672
3	0 h hs-cTnI ≥ 6 & HEAR ≥ 4	38% (563)	99.8 (916/918)	87 (13)	62 (916)	1008	2543
4	0 h hs-cTnI ≥ 13 & HEAR ≥ 4	22% (333)	99.7 (1144/1148)	73 (11)	78 (1144)	913	1821
5	0 h hs-cTnI ≥ 14 & HEAR ≥ 4	21% (316)	99.5 (1159/1165)	60 (9)	79 (1159)	905	1767
6	0 h hs-cTnI ≥ 15 & HEAR ≥ 4	21% (304)	99.5 (1171/1177)	60 (9)	80 (1171)	901	1730
7	0 h hs-cTnI ≥ 20 & HEAR ≥ 4	17% (129)	99.4 (1224/1231)	53 (8)	83 (1224)	878	1560
8	0 h hs-cTnI ≥ 29 & HEAR ≥ 4	13% (197)	99.3 (1275/1284)	40 (6)	87 (1275)	856	1393
9	0 h hs-cTnI ≥ 45 (URL) & HEAR ≥ 4	9% (128)	99.2 (1341/1353)	20 (3)	91 (1341)	828	1177
10	(Counterfactual) HEAR ≥ 4	59% (873)	99.8 (607/608)	93 (15)	41 (607)	1136	3517
11	Observed	59% (879)	99.8 (601/602)	93 (14)	41 (601)	1139	3536

Abbreviations: 0 h hs-cTnI, baseline high-sensitivity cardiac troponin I; HEAR, history, electrocardiogram, age, risk factors; NPV, negative predictive value; URL, upper reference limit.

**Table 5:** Patient non-admitted by standard-of-care and had a 30-day death/MI.

						hs-cTnI (ng/L)				
Patient	Age	Sex	Chief Complaint	Physician Suspicion of Acute Coronary Syndrome	HEAR Score	0 h	Delta 0–1 h	Delta 0–3 h	Disposition	30-Day Event
	34	Male	Fatigue	Low	2	21.69	2.73	3.62	ED	Non-cardiac death
	ICD descriptions									
	Primary cardiomyopathies, congestive heart failure, shortness of breath.									

## Data Availability

The data used in this analysis will be made available upon request.

## Supplementary Materials

The following supporting information can be downloaded at https://www.mdpi.com/article/10.3390/jcdd13070328/s1, Figure S1: Comparison of (A): admission rates and diagnostic accuracy (using 30-day death or MI as the adjudicator); and (B) costs between standard-of-care (i.e., observed) and the best-performing pathway (defined as the lowest-cost pathway maintaining sensitivity compared to standard-of-care) for the final analytic cohort of patients with male sex only (*n* = 880); Figure S2: Comparison of (A): admission rates and diagnostic accuracy (using 30-day death or MI as the adjudicator); and (B) costs between standard-of-care (i.e., observed) and the best-performing pathway (defined as the lowest-cost pathway maintaining sensitivity compared to standard-of-care) for the final analytic cohort of patients with diabetes (*n* = 460); Figure S3: Comparison of (A): admission rates and diagnostic accuracy (using 30-day death or MI as the adjudicator); and (B) costs between standard-of-care (i.e., observed) and the best-performing pathway (defined as the lowest-cost pathway maintaining sensitivity compared to standard-of-care) for the final analytic cohort of patients with obesity (*n* = 708); Figure S4: Comparison of (A): admission rates and diagnostic accuracy (using 30-day death or MI as the adjudicator); and (B) costs between standard-of-care (i.e., observed) and the best-performing pathway (defined as the lowest-cost pathway maintaining sensitivity compared to standard-of-care) for the final analytic cohort of patients aged below 65 years (*n* = 1039); Figure S5: Comparison of (A): admission rates and diagnostic accuracy (using 30-day death or MI as the adjudicator); and (B) costs between standard-of-care (i.e., observed) and the best-performing pathway (defined as the lowest-cost pathway maintaining sensitivity compared to standard-of-care) for the final analytic cohort of patients with presenting hs-cTnI detectable and below 99th percentile (*n* = 1307); Table S1: Components of the modified HEAR score; Table S2: Comparison of admission rates, diagnostic accuracy, and costs by strategy for the final analytic cohort of patients without index AMI, with detectable hs-cTnI, and with no missing data relevant to this analysis (*n* = 1481), using 30-day death or MI as the adjudicator, and with confidence intervals included; Table S3: Comparison of admission rates, diagnostic accuracy (using 30-day death/MI as the adjudicator), and costs between standard-of-care and the best-performing pathway (lowest cost pathway maintaining sensitivity compared to standard-of-care) for each subgroup.
