# Supplementary material for: Clinical and Economic Implications of High-Sensitivity Troponin-Informed Admission Strategies in Non-AMI Chest Pain
Source: J Cardiovasc Dev Dis. 2026 Jul 14;13(7):328. doi: 10.3390/jcdd13070328 (PMC13409828; doi:10.3390/jcdd13070328)

Supplementary Appendix

Clinical and Economic Implications of High-Sensitivity Troponin-Informed Admission Strategies in Non-AMI Chest Pain

Chen et al.

Supplementary Table S1. Components of the modified HEAR score.

	Modified HEAR score	Points
History	High suspicion	2
	Moderate suspicion	1
	Low suspicion	0
ECG	ST-segment deviation	2
	Paced rhythm, LBBB, RBBB, and LVH	1
	Normal or nonspecific changes	0
Age	>65	2
	46-65	1
	45	0
Cardiac risk factors*	Or known CAD	2
	1-2 risk factors	1
	0 risk factors	0

Abbreviations: CAD, coronary artery disease; LBBB, left bundle-branch block; LVH, left ventricular hypertrophy; RBBB, right bundle-branch block.

*Cardiac risk factors: hypertension, diabetes, hyperlipidemia, current smoker, and prior history of myocardial infarction/revascularization/heart failure hospitalization.

Supplementary Table S2. Comparison of admission rates, diagnostic accuracy, and costs by strategy for the final analytic cohort of patients without index AMI, with detectable hs-cTnI, and with no missing data relevant to this analysis (n=1,481), using *30-day* death or MI as the adjudicator, and with confidence intervals included.

	Admission criteria	% admitted	NPV, %	Sensitivity, %	Specificity, %	Cost per patient, $ Scenario 1	Scenario 2
1	0h hs-cTnI ≥4 & HEAR ≥4	46% (43-49%)	99.9 (99.1-100)	93 (68-100)	55 (52-57)	1,057 (1,041-1,072)	2,914 (2,794-3,033)
**2**	**0h hs-cTnI ≥5 (median) & HEAR ≥4**	**41% (38-43%)**	**99.9 (99.2-100)**	**93 (68-100)**	**60 (57-62)**	**1,025 (1,009-1,040)**	**2,672 (2,554-2,790)**
3	0h hs-cTnI ≥6 & HEAR ≥4	38% (36-41%)	99.8 (99.0-99.9)	87 (60-98)	62 (60-65)	1,008 (992-1,023)	2,543 (2,427-2,660)
4	0h hs-cTnI ≥13 & HEAR ≥4	22% (20-25%)	99.7 (99.0-99.9)	73 (45-92)	78 (76-80)	913 (899-926)	1,821 (1,720-1,921)
5	0h hs-cTnI ≥14 (3rd quartile) & HEAR ≥4	21% (19-24%)	99.5 (98.8-99.8)	60 (32-84)	79 (77-81)	905 (892-918)	1,767 (1,669-1,866)
6	0h hs-cTnI ≥15 & HEAR ≥4	21% (18-23%)	99.5 (98.8-99.8)	60 (32-84)	80 (78-82)	901 (888-913)	1,730 (1,632-1,827)

Supplementary Table S2. Continued.

	Admission criteria	% admitted	NPV, %	Sensitivity, %	Specificity, %	Cost per patient, $ Scenario 1	Scenario 2
7	0h hs-cTnI ≥20 & HEAR ≥4	17% (15-19%)	99.4 (98.7-99.7)	53 (27-79)	83 (81-85)	878 (866-890)	1,560 (1,470-1,650)
8	0h hs-cTnI ≥29 & HEAR ≥4	13% (12-15%)	99.3 (98.6-99.6)	40 (16-68)	87 (85-89)	856 (845-867)	1,393 (1,311-1,475)
9	0h hs-cTnI ≥45 (URL) & HEAR ≥4	7% (6-8%)	99.1 (98.4-99.5)	20 (4-48)	91 (90-93)	828 (819-837)	1,177 (1,109-1,245)
10	(Counterfactual) HEAR≥4	53% (50-55%)	99.8 (98.8-100)	93 (68-100)	41 (39-44)	1,136 (1,121-1,152)	3,517 (3,399-3,635)
**11**	**Observed**	**54% (52-57%)**	**99.8 (98.8-100)**	**93 (68-100)**	**41 (38-44)**	**1,139 (1,123-1,154)**	**3,536 (3,418-3,654)**

Abbreviations: 0h hs-cTnI, baseline high-sensitivity cardiac troponin I; HEAR, History, Electrocardiogram, Age, Risk factors; NPV, negative predictive value; URL, upper reference limit.

Supplementary Table S3. Comparison of admission rates, diagnostic accuracy (using 30-day death/MI as the adjudicator), and costs between standard-of-care and the best-performing pathway (lowest cost pathway maintaining sensitivity compared to standard-of-care) for each subgroup.

% admitted (n/N)		NPV, % (n/N)		Sensitivity, % (n/N)		Specificity, % (n/N)		$/pt, Scenario 1		$/pt, Scenario 2	
**Subgroup: male sex only; Best-performing pathway: 0h hs-cTnI ≥6 & HEAR ≥4 (HEAR+6)**											
**SOC**	**HEAR+6**	**SOC**	**HEAR+6**	**SOC**	**HEAR+6**	**SOC**	**HEAR+6**	**SOC**	**HEAR+6**	**SOC**	**HEAR+6**
62 (549/880)	41 (363/880)	99.7 (330/331)	99.8 (516/517)	92 (11/12)	92 (11/12)	38 (330/868)	59 (516/868)	1,157	1,028	3,677	2,694
**Subgroup: patients with diabetes; Best-performing pathway: 0h hs-cTnI ≥6 & HEAR ≥4**											
**SOC**	**HEAR+6**	**SOC**	**HEAR+6**	**SOC**	**HEAR+6**	**SOC**	**HEAR+6**	**SOC**	**HEAR+6**	**SOC**	**HEAR+6**
69 (318/460)	52 (238/460)	100.0 (142/142)	100.0 (142/142)	100.0 (9/9)	100.0 (9/9)	31 (142/451)	49 (222/451)	1,199	1,092	3,991	3,182
**Subgroup: patients with obesity (n=708); Best-performing pathway: 0h hs-cTnI ≥13 & HEAR ≥4**											
**SOC**	**HEAR+13**	**SOC**	**HEAR+13**	**SOC**	**HEAR+13**	**SOC**	**HEAR+13**	**SOC**	**HEAR+13**	**SOC**	**HEAR+13**
60 (423/708)	22 (156/708)	99.6 (284/285)	99.8 (551/552)	83 (5/6)	83 (5/6)	40 (284/702)	78 (551/702)	1,141	1,008	3,554	1,800
**Subgroup: patients aged below 65 years (n=1,039); Best-performing pathway: 0h hs-cTnI ≥6 & HEAR ≥4**											
**SOC**	**HEAR+6**	**SOC**	**HEAR+6**	**SOC**	**HEAR+6**	**SOC**	**HEAR+6**	**SOC**	**HEAR+6**	**SOC**	**HEAR+6**
55 (567/1,039)	29 (298/1,039)	99.8 (471/472)	99.9 (740/741)	86 (6/7)	86 (6/7)	46 (471/1,032)	72 (740/1,032)	1,109	951	3,313	2,109
**Subgroup: patients with presenting hs-cTnI detectable and below 99th percentile (n=1,307); Best-performing pathway: 0h hs-cTnI ≥6 & HEAR ≥4**											
**SOC**	**HEAR+6**	**SOC**	**HEAR+6**	**SOC**	**HEAR+6**	**SOC**	**HEAR+6**	**SOC**	**HEAR+6**	**SOC**	**HEAR+6**
57 (747/1,307)	33 (435/1,307)	99.8 (559/560)	99.8 (870/872)	92 (11/12)	83 (10/12)	43 (559/1,295)	67 (870/1,295)	1,125	979	3,433	2,323

Supplementary Figure S1. Comparison of (A): admission rates and diagnostic accuracy (using 30-day death or MI as the adjudicator); and (B) costs between standard-of-care (i.e., observed) and the best-performing pathway (defined as the lowest-cost pathway maintaining sensitivity compared to standard-of-care) for the final analytic cohort of patients with male sex only (n=880).

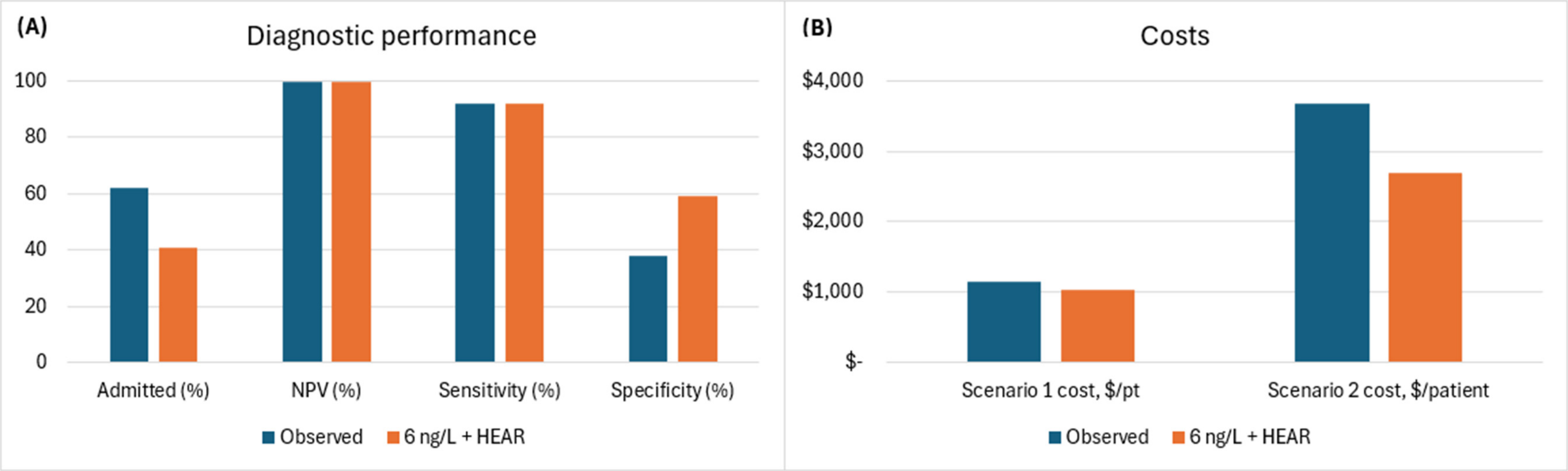

Supplementary Figure S2. Comparison of (A): admission rates and diagnostic accuracy (using 30-day death or MI as the adjudicator); and (B) costs between standard-of-care (i.e., observed) and the best-performing pathway (defined as the lowest-cost pathway maintaining sensitivity compared to standard-of-care) for the final analytic cohort of patients with diabetes (n=460).

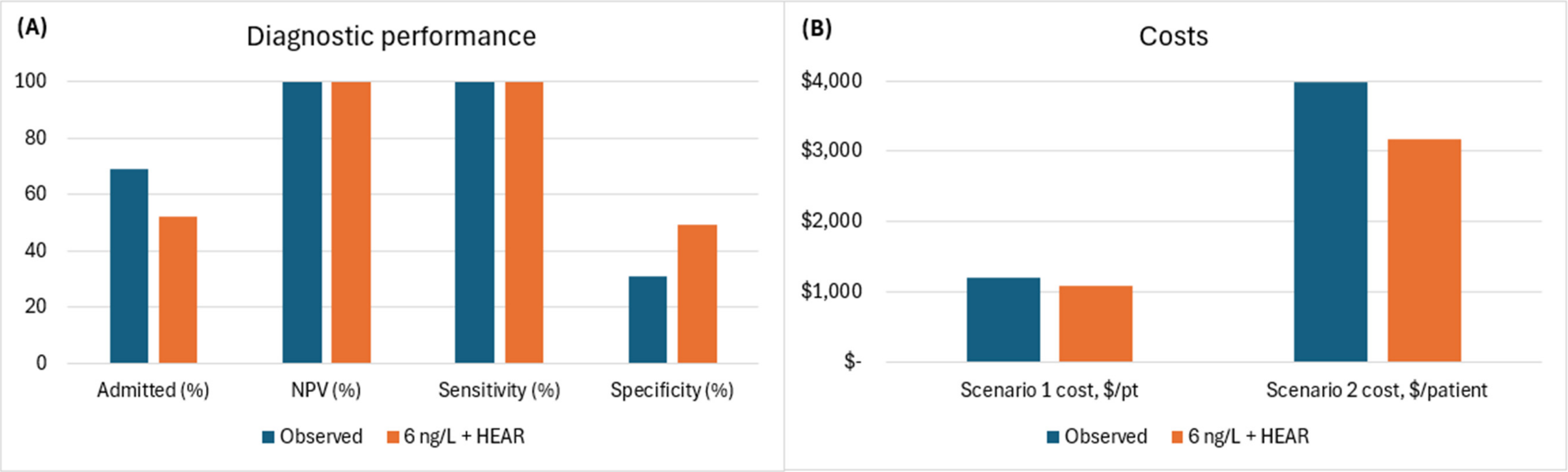

Supplementary Figure S3. Comparison of (A): admission rates and diagnostic accuracy (using 30-day death or MI as the adjudicator); and (B) costs between standard-of-care (i.e., observed) and the best-performing pathway (defined as the lowest-cost pathway maintaining sensitivity compared to standard-of-care) for the final analytic cohort of patients with obesity (n=708).

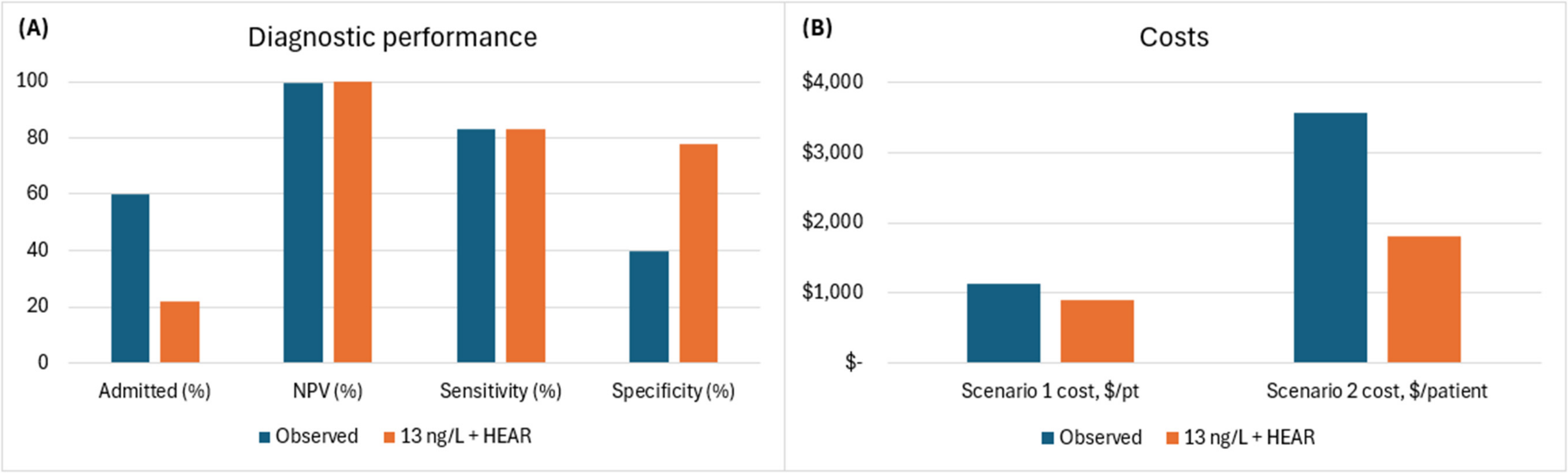

Supplementary Figure S4. Comparison of (A): admission rates and diagnostic accuracy (using 30-day death or MI as the adjudicator); and (B) costs between standard-of-care (i.e., observed) and the best-performing pathway (defined as the lowest-cost pathway maintaining sensitivity compared to standard-of-care) for the final analytic cohort of patients aged below 65 years (n=1,039).

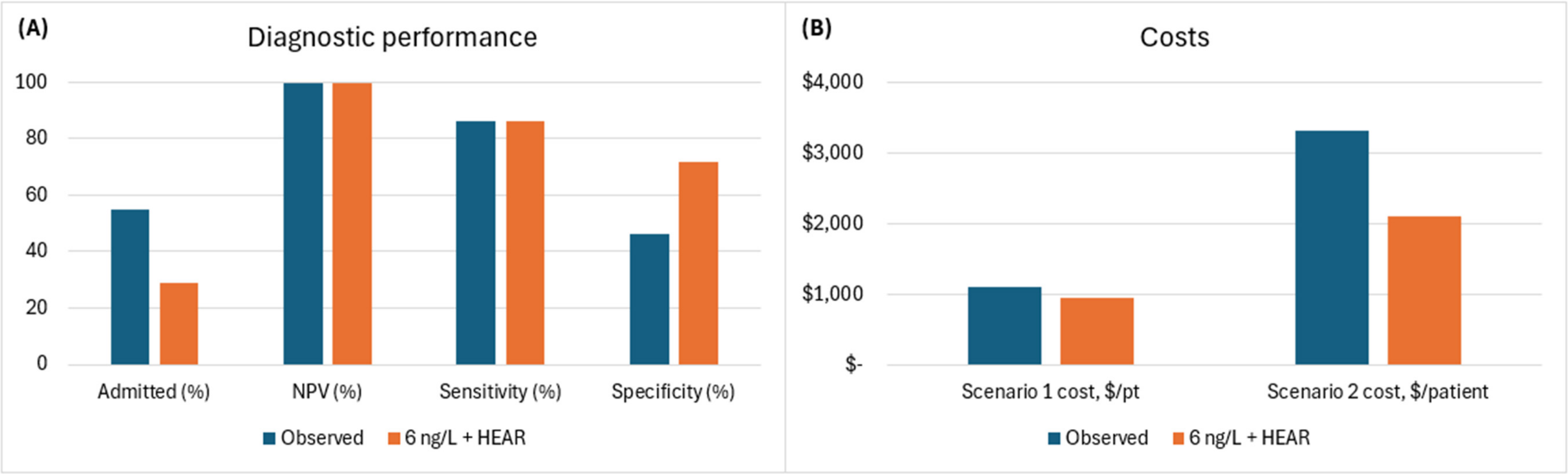

Supplementary Figure S5. Comparison of (A): admission rates and diagnostic accuracy (using 30-day death or MI as the adjudicator); and (B) costs between standard-of-care (i.e., observed) and the best-performing pathway (defined as the lowest-cost pathway maintaining sensitivity compared to standard-of-care) for the final analytic cohort of patients with presenting hs-cTnI detectable and below 99th percentile (n=1,307).

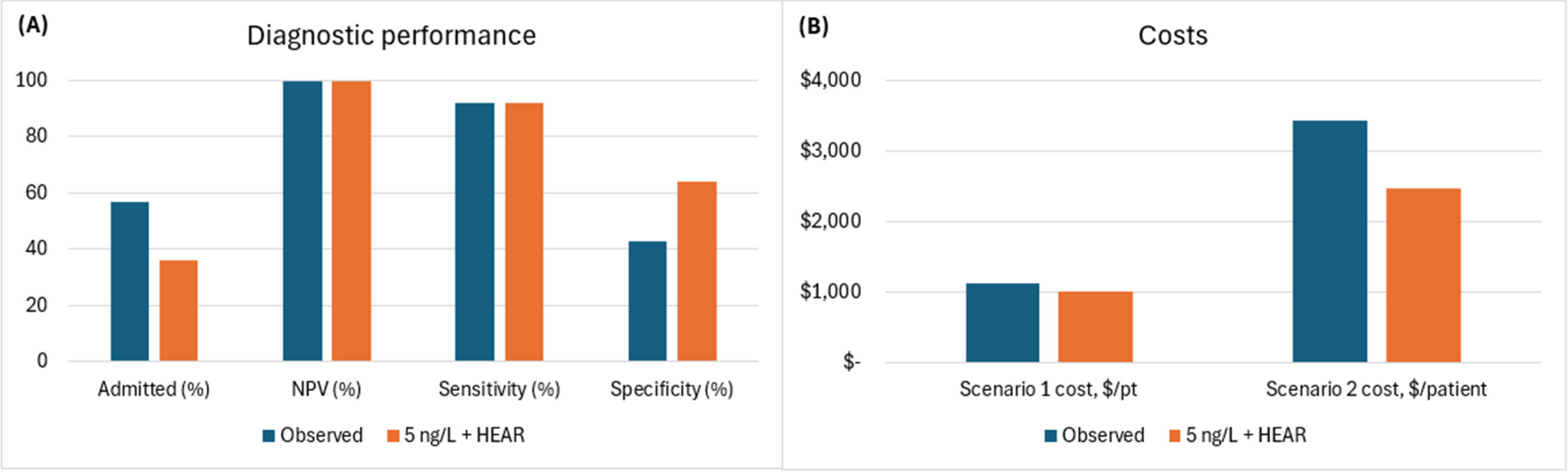

Supplement: Supplementary file 1 [file jcdd-13-00328-s001.zip › jcdd-4346542-supplementary.pdf]
